# Local Inflammation in Fracture Hematoma: Results from a Combined Trauma Model in Pigs

**DOI:** 10.1155/2015/126060

**Published:** 2015-01-28

**Authors:** K. Horst, D. Eschbach, R. Pfeifer, S. Hübenthal, M. Sassen, T. Steinfeldt, H. Wulf, S. Ruchholtz, H. C. Pape, F. Hildebrand

**Affiliations:** ^1^Department of Orthopedic Trauma and Harald Tscherne Research Laboratory, University Hospital Aachen, Pauwelsstraße 30, 52074 Aachen, Germany; ^2^Department of Hand, Traumatology and Reconstructive Surgery, University Hospital Marburg, Marburg, Germany; ^3^Department of Anesthesiology, University Hospital Marburg, Marburg, Germany

## Abstract

*Background*. Previous studies showed significant interaction between the local and systemic inflammatory response after severe trauma in small animal models. The purpose of this study was to establish a new combined trauma model in pigs to investigate fracture-associated local inflammation and gain information about the early inflammatory stages after polytrauma.* Material and Methods*. Combined trauma consisted of tibial fracture, lung contusion, liver laceration, and controlled hemorrhage. Animals were mechanically ventilated and under ICU-monitoring for 48 h. Blood and fracture hematoma samples were collected during the time course of the study. Local and systemic levels of serum cytokines and diverse alarmins were measured by ELISA kit.* Results*. A statistical significant difference in the systemic serum values of IL-6 and HMGB1 was observed when compared to the sham. Moreover, there was a statistical significant difference in the serum values of the fracture hematoma of IL-6, IL-8, IL-10, and HMGB1 when compared to the systemic inflammatory response. However a decrease of local proinflammatory concentrations was observed while anti-inflammatory mediators increased.* Conclusion*. Our data showed a time-dependent activation of the local and systemic inflammatory response. Indeed it is the first study focusing on the local and systemic inflammatory response to multiple-trauma in a large animal model.

## 1. Introduction

Multiple trauma represents one of the most frequent causes of death particularly in the younger population [1, 2]. Besides these fatal consequences, trauma also accounts for impaired long-term outcome due to disabilities, which are mainly caused by extremity injuries [3, 4].

Previous clinical and experimental studies showed that multiple trauma is associated with a significantly longer fracture healing time and a higher incidence of nonunions in comparison to isolated fractures [5]. Furthermore, it showed that overwhelming local and systemic inflammatory responses with an associated negative influence on downstream processes of bone repair are a potential pathomechanism for this impaired fracture healing [6–9].

Despite this significant interaction between the inflammatory response and fracture healing, few studies focus on the interaction between local and systemic inflammatory response after trauma. These studies either focused on isolated fractures [10–13] or used small animal models with the well-known limitations of translational significance [14–16]. On the other hand, large animal models were established in order to better mimic the human situation [17, 18], as the majority of these models were limited by either focus on isolated fractures, a very short posttraumatic observation period (2–6 h), or the fact that animals were extubated and observed in an awake state, which also does not represent the clinical situation after multiple trauma [17, 18].

The purpose of the study was to establish a combined trauma model (chest, abdominal injury and controlled hemorrhagic shock) including extremity injury and to investigate the early kinetics of the local immunologic response in the fracture hematoma in this clinically relevant porcine model with mechanical ventilation and intensive care treatment over the entire study period of 48 hours.

## 2. Material and Methods

### 2.1. Animal Care

Approval of the Regional Ethics Committee and the Animal Welfare Authority was obtained. In total, 20 male pigs (*Sus scrofa*, 35 ± 5 Kg/3 months old) were used in this experiment. We used 5 pigs as the sham group and 15 pigs as the study arm.

### 2.2. General Instrumentation and Anesthesia

After a fasting period of 12 hours, anesthesia was induced and pigs were intubated (7,5 ch tube). Ventilation was obtained in BiPAP mode using a defined volume of 6–8 mL/kg/BW with a FiO2 of 30% and a PEEP of 5 mmHg, adjusted by capnometria targeting 45–55 mmHg (Draeger, Evita, Danvers, MA, USA). Vital signs were monitored by electrocardiographic recording (ECG) and ECG-synchronized pulse oximetry. General anesthesia was maintained by application of Propofol, Sufentanil, and Midazolam during the entire study period. Fluids were additionally administered by continuous crystalloid infusion over the entire period (2 mL kg^−1^ h^−1^). Single shot antibiosis was administered before the interventions (cefuroxime 1.5 g). Arterial pulse contour cardiac output (PiCCO, Pulsion Medical Systems, Germany) catheter (left femoral arteria), a two-lumen hemodialysis catheter (Arrow International, Germany) (left femoral vein), a central venous catheter (Arrow International, Germany) (right jugular vein), and a suprapubic catheter were placed under sterile conditions. Operative tracheotomy was performed and a shortened 7,5 ch endotracheal tube was placed.

### 2.3. Induction of Combined Trauma and Hemorrhage

Trauma consisted of a tibia fracture induced by placing the lower leg into a drop-weight gadgetry. A 20 kg plumb-cuboid was dropped from a height of 100 cm. Afterwards a cast was applied. Blunt thoracic trauma was induced by applying a panel of 1 cm thickness to the right dorsal, lower chest. A bolt was shot onto this panel using cattle killing cartridges (9 × 17; Dynamit Nobel AG, Troisdorf, Germany) simulating blunt lung contusion. The shot was applied while the lungs of the animals were inflated and inspiratory O_2_ was defined at 21% during the trauma period, simulating the ambient air. Next, a midline-laparotomy was performed and the right upper liver lobe was explored. Using a sharp, custom-made, four-edged scalpel a penetrating abdominal injury was induced. After a short period of uncontrolled bleeding (approximately 30 seconds), liver packing was carried out with seven sterile packs of the same size. Laceration associated bleeding was assessed macroscopically after 24 h, sterile packs were replaced, and the abdomen was surgically closed again. The evaluation and documentation were performed by an experienced surgeon. After hepatic packing pressure-controlled and volume-limited hemorrhagic shock was induced by withdrawing blood until a middle arterial pressure (MAP) of 30 ± 5 mmHg was reached. In this context, a maximum of 45% of total blood volume was drawn from the left femoral vein. The shed blood was abolished. Hemorrhagic shock was maintained for 90 min. Thereafter, the animals were resuscitated using crystalloid fluids in a volume of four times the shed blood volume over a period of 1 hour.

Sham animals and trauma animals were identically instrumented and received the same anesthetic and intensive care procedures. Respiratory and blood gas parameters were monitored and adjusted if necessary to keep parameters on a physiologic level at baseline. Compared to trauma animals, sham animals were not subjected to any injury or hemorrhage. In both groups (trauma animals and sham animals), blood samples were collected at the same time points.

### 2.4. Data Collection

Full blood samples were drawn exactly before induction of trauma (0 h), after trauma and resuscitation (2.5 h), and during observation period at 14 h, 24 h, and 48 h using monovettes (SARSTEDT AG & Co, Germany). Fracture hematoma was extracted under sterile conditions by puncturing the fracture zone. Hematoma was collected in an EDTA monovette (SARSTEDT AG & Co, Germany). After centrifugation, serum was removed and stored at −80°C for further analysis. After an observation period of 48 hours the animals were sacrificed. Data was collected using a Filemaker Database (FilmakerPro5.0, Filemaker Inc.); access was limited by password protection. Security setup was done every 48 h.

### 2.5. Laboratory Evaluation

Serum levels of Interleukin- (IL-) 6, IL-8, and IL-10, High-Mobility-Group-Protein B1 (HMGB1), and Heat Shock Protein (HSP) 70 were analyzed using samples taken before trauma (0 h), after trauma and resuscitation (2.5 h), and at 14 h, 24 h, and 48 h after beginning of the study. Samples of fracture hematoma were analyzed using material from time points at 14 h, 24 h, and 48 h.

IL-6, IL-8, and IL-10, HMGB1, and HSP70 were analyzed from serum samples using ELISA kits (IL-6, -IL-8, and IL-10: R&D systems, USA; HMGB1: IBL International GmbH, Germany; HSP70: USCN Life Science Inc., China) according to the manufacturer protocol. Fracture hematoma was analyzed using the same kits. All hematoma samples were centrifugalized again at 4°C before usage. Referring to higher concentrations, all fracture hematoma samples were diluted (IL-6: 1 : 10, IL-8: 1 : 4, IL-10: 1 : 4, HMGB1: 1 : 10, and HSP70: 1 : 10).

### 2.6. Statistics

Statistics were done with Excel (Microsoft 2010) and SPSS (Version 21.0.0.0) using KS-test for normal distribution, Student's *t*-test for means (illustrated as mean ± SD), and Wilcoxon rank sum test for statistical significance (*P* ≤ 0.05). Results are demonstrated as mean and standard deviation (±SD).

## 3. Results

### 3.1. Survival

The traumatized animals showed a mortality of 13% (*n* = 2) while all animals of the sham group survived. Cause of death was cardiac arrest within the first 12 h of investigation due to ventricular fibrillation. Cardiopulmonary resuscitation was necessary in 26% (*n* = 4) of all traumatized pigs, which was successful in 50% of the cases.

### 3.2. Hemodynamic and Physiologic Parameters

An average of 43.8% (SD ± 5.97) of total blood volume (TBV) was withdrawn for shock induction. Mean arterial pressure was 29 mmHg (SD ± 7) mm/Hg and maximal heart rate was 197 beats/min (SD ± 28) during the shock period in traumatic pigs. Compared to sham animals, hemoglobin (Hb) was significantly lower in the trauma group after reperfusion and remained lower over the entire study period (Table 1). Lactate (Lac) levels were significantly elevated after trauma and reperfusion (Table 1). Base excess (BE) significantly decreased during the trauma period (Table 1). The animals that died during investigation all showed BE levels ≤−8 mmol/L.

### 3.3. Cytokines

#### 3.3.1. Systemic Concentrations

**Figure 1:**
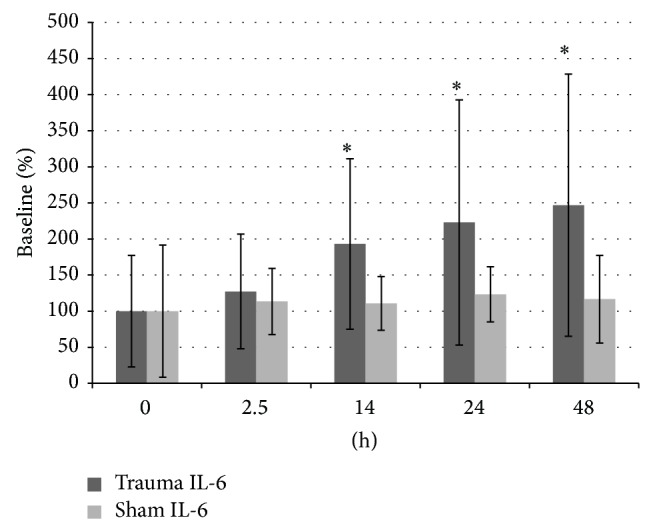
Serum IL-6 levels, ∗ = *P* < 0.05 compared to sham group.

Serum levels of IL-6 increased over time in the traumatic group and showed highest level at the end of the observation period (0 h versus 48 h: *P* = 0.010). Significantly higher levels of IL-6 were seen after 14 h (*P* = 0.014), 24 h (*P* = 0.019), and 48 h (*P* = 0.048) when compared to sham group (Figure 1). In contrast, there was no statistical significant difference in the serum levels of IL-8 and IL-10 over the study time when compared to the sham group (data not shown).

#### 3.3.2. Local Concentrations

**Figure 2:**
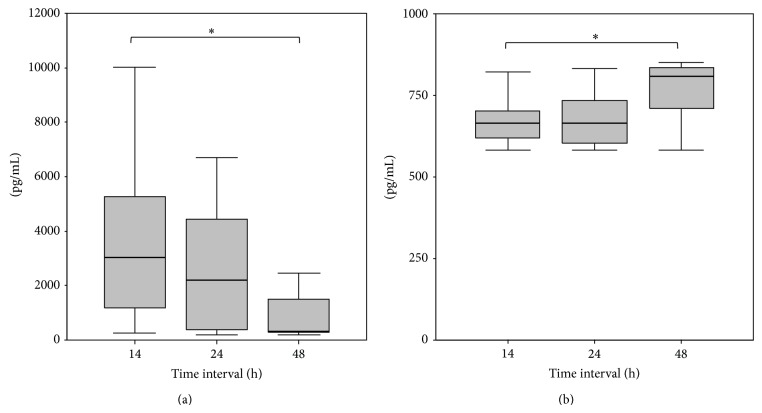
(a) Decreasing fracture hematoma concentration of IL-6, ∗ = *P* < 0.05 between 14 h and 48 h. (b) Increasing fracture hematoma concentration of IL-8, ∗ = *P* < 0.05 between 14 h and 48 h.

Serum levels of IL-6, IL-8, and IL-10 were significantly higher in the fracture hematoma when compared to the systemic serum levels at every time point of the observation period (Table 2). IL-6 levels in the fracture hematoma were highest at 14 h and significantly decreased in the further course (14 h versus 48 h: *P* = 0.028). IL-8 concentrations showed a significant increase over time (14 h versus 48 h: *P* < 0.05). IL-10 concentrations did not show any significant changes in kinetics although a minimal increase was observed (Figures 2(a) and 2(b)).

### 3.4. Alarmins

#### 3.4.1. Systemic Concentrations

**Figure 3:**
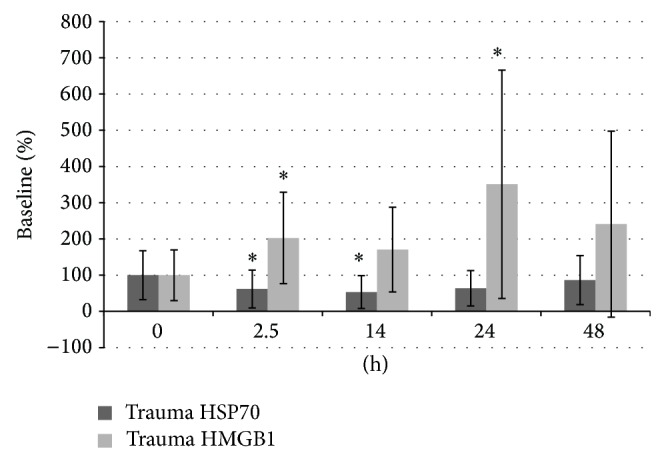
HMGB1 & HSP70 serum concentrations, ∗ = *P* < 0.05 compared to baseline values and sham animals.

After 2.5 h HMGB1 serum concentrations showed a statistical significant increase after 2.5 h after injury when compared to the baseline concentrations (*P* = 0.028) and when compared to the sham group at the respective time point (*P* = 0.013) (Figure 3). After resuscitation, HMGB1 levels decreased with a secondary peak at 24 h with significant differences compared to baseline values (*P* = 0.01) and compared to the sham group (*P* = 0.04). After trauma induction, HSP70 levels decreased (2.5 h: *P* = 0.002; 14 h: *P* = 0.021; 24 h: *P* = 0.006) with significant reduced levels compared to the sham group at time points 2.5 h (*P* = 0.02) and 14 h (*P* = 0.026) (Figure 3).

#### 3.4.2. Local Concentrations

**Figure 4:**
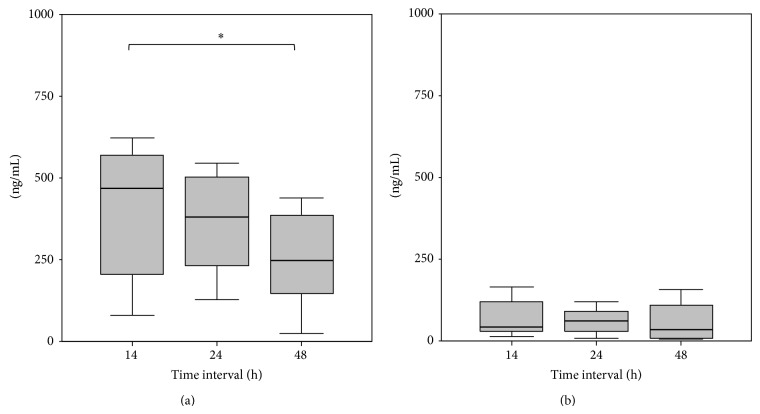
(a) Decreasing fracture hematoma concentrations of HMBG1, ∗ = *P* < 0.05 between 14 h and 48 h. (b) Stable fracture hematoma concentrations of HSP70, ∗ = *P* < 0.05 between 14 h and 48 h.

The fracture hematoma samples showed that HMGB1 significantly decreased over time (14 h versus 48 h: *P* = 0.047) while HSP70 concentrations remained unchanged (Figures 4(a) and 4(b)). Furthermore, HMGB1 concentrations in fracture hematoma were significantly higher than in the systemic serum concentrations, while HSP70 fracture hematoma concentrations only tended to be higher when compared to the systemic serum concentrations (Table 2).

## 4. Discussion

Previous studies showed that early local inflammatory response plays a major role in the process of fracture healing following trauma [10, 11, 19]. Yet, this field is still vague and unclear as most of the work was made on small animal models either with limited observation time or under condition that does not closely mimic the clinical situation [16, 20–23]. Therefore, we investigated the local and systemic inflammatory response in porcine animal model of polytrauma, as pigs which respond to trauma resemble humans [24, 25].

The main results might be summarized as follows.

- This newly established model is clinically relevant reflected by a significant systemic inflammatory response (IL-6 and HMGB1).
- Compared to systemic values, local concentrations (fracture hematoma) of pro- and anti-inflammatory mediators were significantly higher and showed earlier increase.
- Concentrations of local proinflammatory mediators decreased over time while levels of anti-inflammatory mediators increased.

### 4.1. Clinical Relevance of the Presented Model

The kinetics of systemic cytokine levels after severe trauma and their significance for the posttraumatic course have been described in numerous studies [26–30]. In concordance with the well-described systemic increase of IL-6 concentrations after trauma [28, 31] we also observed a significant increase of systemic IL-6 levels, which underlines the clinical relevance of our model. Also in accordance with previously published data from a porcine model of combined trauma [32] we did not find significant changes of systemic IL-8 or IL-10 concentrations.

Our results matched what other groups found in regard to the systemic increase of HMGB1 after trauma [33–35]. However, we also found evidence for a second peak in the later posttraumatic course, which was more pronounced when compared to the initial peak. Compared to other studies that describe HMGB1 as continuously increasing, only Yao and Lin mentioned this posttraumatic dichotomous pattern so far [36]. Further studies must investigate which factors contribute to the observed kinetics of systemic HMBG1 levels [37].

Our results showed that systemic HSP70 concentration decreased in the early posttraumatic phase as other groups showed for different organ systems earlier [38, 39]. In this context Kirchhoff et al. [40] concluded that immediate hyperactivation of circulating monocytes after severe trauma is rapidly followed by a substantial paralysis of cell function which most likely is also the reason for the observed findings in our study. Baker et al. in contrast [32] described a posttraumatic increase of HSP70 concentrations. However, the applied models of trauma in their investigation were less severe and observation time was limited to 300 minutes only. Furthermore female pigs were included although it is well known that female sex hormones have a beneficial effect on HSP expression [41]. Additional studies must uncover the role of HSP subgroups in the multiple injured.

### 4.2. Fracture Hematoma Concentrations of Cytokines and Alarmins

In accordance with our results in this study, previous work in a small animal model of isolated femoral fracture showed that IL-6 increases in the early phase after trauma and later on decreases in the fracture site [14, 42]. In this study an IL-6-induced upregulation of the suppressor of cytokine signaling-3 (SOCS-3) has been suggested as a possible mechanism for the reduction of local IL-6 concentrations [14]. In general, IL-6 has been shown to play a significant role in the process of fracture healing [43, 44]. In this context, a significant increase of fracture callus and earlier bone union was observed after combined application of parathyroid hormone and IL-6 compared to isolated administration of parathyroid hormone [15]. However, de Benedetti et al. showed that overexpression of IL-6 resulted in severe osteopenia with reduced osteoblast and increased osteoclast numbers and activity. Accordingly, Recknagel et al. demonstrated that a systemic inflammatory response with increased IL-6 concentrations after blunt chest trauma might impair bone healing [45]. Therefore it was suggested that effects of IL-6 on bone cells seem to be concentration dependent [46].

In this study we measured some of the anti-inflammatory mediators like IL-8 and IL-10 locally and systemically as they play central role in the fracture healing process [10]. Our data showed increased levels of IL-8 from the fracture hematoma samples, which were significantly higher compared to the systemic levels. Accordingly, high local levels were found in fracture hematoma in a previous clinical study which underlines the importance of IL-8 in the process of bone healing [10]. Furthermore, our results showed significant early elevation of IL-10 in the fracture hematoma. Accordingly, Hauser et al. found significantly increased IL-10 levels in fracture hematomas in the early phase after trauma whereas lower levels were observed in the later period (>24 h). The authors therefore assumed that immunosuppressive cytokines accumulating within the fracture/soft-tissue hematomas might potentially be involved in the suppression of cell mediated immunity in trauma patients. Interestingly, comparable to our results patients plasma concentrations did not differ from the control group [12].

From their results in small animal model of HMGB1 knockout mice, Taniguchi et al. found evidence that HMGB1 is of major importance for tissue repair and bone organization after trauma [47]. Despite this potential significant role of HMGB1 in the process of posttraumatic tissue organization, information on local concentrations of alarmins after fractures is sparse. The high local concentrations of HMGB1, found in our study, support the assumption that HMGB1 plays an important role in tissue healing during the early posttraumatic phase. Also for HSPs regulatory mechanisms on bone healing have been found. HSP27 has effects on osteoblast function [48, 49], whereas HSP70 has stimulatory effects on bone morphogenetic proteins (BMP) secretion [50]. However, there is also a lack of knowledge about the kinetics of local HSP in fracture hematoma. To the best of our knowledge, our study is the first that describes an increase of anti-inflammatory HSP70 in fracture hematoma. Thus, there is evidence that over time there is a change from a proinflammatory milieu towards an anti-inflammatory milieu in fracture hematoma which might significantly influence tissue healing.

### 4.3. Limitations

The purpose of our study was to gain knowledge and senses about the kinetics of the local inflammatory response in a clinically relevant, large animal model of combined trauma. Our data do not permit conclusions on the molecular mechanisms that regulate local or systemic inflammatory response. Furthermore, no assumptions on the interaction with osteo- and chondrogenesis can be drawn. Furthermore individual immunologic responses must be considered. Research regarding this field is ongoing. Finally our results need to be compared to a group with isolated extremity injury to gain further information. This will be the focus of a follow-up study.

## 5. Conclusion

To the best of our knowledge, this is the first study that describes chronologic data of locally active inflammatory mediators and quantifies molecules within fracture hematoma in a combined trauma model. Although a causation to systemically circulating mediators cannot be drawn from this model, it might be suggested that fracture/soft-tissue hematomas appear to be an important source of systemic inflammatory mediators after severe trauma. Conversely, significantly increased local levels of inflammatory mediators after combined trauma might also influence fracture healing. Based on the results of this study, further studies of our group will focus on the role of inflammatory mediators in the repairing process of injured tissue and their role in the systemic process of responding to trauma.

## Figures and Tables

**Table 1:** Laboratory parameters: hemoglobin (Hb), base excess (BE), lactate (Lac), and not significant (n.s.).

	0 h	2.5 h	14 h	24 h	48 h
Hb trauma (mg/dL)	91.27 ± 8.35	46.36 ± 8.03	54.38 ± 7.98	54.62 ± 11.07	48.23 ± 6.43
Hb sham (mg/dL)	97.6 ± 13.61	90.4 ± 4.56	88.2 ± 5.85	84.8 ± 4.6	71.2 ± 9.96
*P* value	**n.s.**	**<0.001**	**<0.001**	**<0.001**	**<0.001**

BE trauma (mmol/L)	4.09 ± 2.66	2.14 ± 3.75	6.38 ± 0.89	5.73 ± 2.89	6.28 ± 1.52
BE sham (mmol/L)	5.58 ± 1.96	6.44 ± 1.64	6.03 ± 0.9	5.8 ± 1.19	7.28 ± 2.21
*P* value	**n.s.**	**0.026**	**n.s.**	**n.s.**	**n.s.**

Lac trauma (mmol/L)	0.89 ± 0.39	2.64 ± 2.14	0.66 ± 0.21	0.9 ± 0.47	1.08 ± 1.25
Lac sham (mmol/L)	1.26 ± 0.46	0.84 ± 0.15	0.52 ± 0.08	0.44 ± 0.11	0.56 ± 0.18
*P* value	**n.s.**	**0.008**	**n.s.**	**0.05**	**n.s.**

**Table 2:** Comparison of serum and fracture hematoma concentrations on 14 h, 24 h, and 48 h.

	14 h	24 h	48 h
Serum IL-6 (pg/mL)	62.35 ± 38.12	71.95 ± 54.76	79.69 ± 58.59
Hematoma IL-6 (pg/mL)	3631.65 ± 2992.31	2725.91 ± 2370.57	1120.99 ± 1561.31
*P* value	**0.002**	**0.002**	**0.002**

Serum IL-8 (pg/mL)	148.90 ± 25.85	161.78 ± 32.92	139.71 ± 34.44
Hematoma IL-8 (pg/mL)	658.29 ± 84.11	676.30 ± 181.66	877.69 ± 491.47
*P* value	**0.002**	**0.002**	**0.002**

Serum IL-10 (pg/mL)	64.56 ± 41.83	64.55 ± 37.31	59.50 ± 30.24
Hematoma IL-10 (pg/mL)	245.57 ± 131.97	262.10 ± 163.15	271.47 ± 193.26
*P* value	**0.004**	**0.002**	**0.003**

Serum HMGB1 (ng/mL)	2.2 ± 1.54	4.8 ± 3.9	3.01 ± 3.21
Hematoma HMGB1 (ng/mL)	573.5 ± 504.35	351.89 ± 152.79	246.79 ± 135
*P* value	**0.008**	**0.005**	**0.003**

Serum HSP70 (ng/mL)	47.04 ± 40.07	56.06 ± 42.95	75.96 ± 59.36
Hematoma HSP70 (ng/mL)	87.17 ± 95.82	84.75 ± 99.81	130.61 ± 256.02
*P* value	**n.s.**	**n.s.**	**n.s.**

## Advances in Knowledge

To our knowledge, this is the first study describing and discussing systemic and local (fracture hematoma) inflammatory response in a combined large animal model giving chronologic data of locally active inflammatory mediators in regard to extremity fracture during early posttraumatic stages.
